# Microbial single-cell applications under anoxic conditions

**DOI:** 10.1128/aem.01321-24

**Published:** 2024-09-30

**Authors:** Ciara Keating, Kerstin Fiege, Martijn Diender, Diana Z. Sousa, Laura Villanueva

**Affiliations:** 1 Department of Engineering, Durham University, Durham, United Kingdom; 2 Department of Marine Microbiology and Biogeochemistry, Royal Netherlands Institute for Sea Research (NIOZ), Den Burg, the Netherlands; 3 Laboratory of Microbiology, Wageningen University & Research, Wageningen, the Netherlands; 4 Centre for Living Technologies, Alliance TU/e, WUR, UU, UMC Utrecht, Utrecht, the Netherlands; 5 Department of Biology, Utrecht University, Utrecht, the Netherlands; Georgia Institute of Technology, Atlanta, Georgia, USA

**Keywords:** anoxic cultivation, single-cell, anaerobes

## Abstract

The field of microbiology traditionally focuses on studying microorganisms at the population level. Nevertheless, the application of single-cell level methods, including microfluidics and imaging techniques, has revealed heterogeneity within populations, making these methods essential to understand cellular activities and interactions at a higher resolution. Moreover, single-cell sorting has opened new avenues for isolating cells of interest from microbial populations or complex microbial communities. These isolated cells can be further interrogated in downstream single-cell “omics” analyses, providing physiological and functional information. However, applying these methods to study anaerobic microorganisms under *in situ* conditions remains challenging due to their sensitivity to oxygen. Here, we review the existing methodologies for the analysis of viable anaerobic microorganisms at the single-cell level, including live-imaging, cell sorting, and microfluidics (lab-on-chip) applications, and we address the challenges involved in their anoxic operation. Additionally, we discuss the development of non-destructive imaging techniques tailored for anaerobes, such as oxygen-independent fluorescent probes and alternative approaches.

## INTRODUCTION

Some of the world’s most fascinating ecosystems are characterized by anoxic environments, devoid of molecular oxygen. The absence of oxygen imposes energetic constraints on microorganisms in these environments, requiring the utilization of alternative electron acceptors with lower redox potentials (1, 2). Anoxic settings are found in soils, sediments, rice paddy fields, the gut, and the deep sea. Microorganisms living in anoxic environments can exhibit either anaerobic (conducted in the absence of measurable oxygen) or facultative anaerobic metabolism. The term “anaerobic” is commonly interchanged by anoxic, while anoxic is not linked to metabolisms and refers more generally to an environment or culture setting which is oxygen-free or with oxygen concentrations below the detection level. Environments can also be “hypoxic” referring to the presence of low or depleted oxygen concentration (<21% O_2_). The terms “micro-oxic” or “micro-anoxic” are also occasionally used for similar environments. In contrast, oxic environments are those which contain oxygen (the term aerobic will be used throughout this manuscript).

Anaerobic metabolism relies on the presence of alternative electron acceptors to oxygen, while facultative anaerobes are more versatile and can also utilize oxygen when available. Some anaerobes can tolerate oxygen (aerotolerants), or require low levels of oxygen (microaerophiles), while others are extremely sensitive to its presence (strict anaerobes); for example, most methanogenic (methane producing) archaea are notoriously sensitive to oxygen concentrations (poisoned at >5 ppm O_2_) (3).

Anaerobes play key roles in organic matter degradation, biogeochemical cycling, and even contribute or alleviate climate change by the production or consumption of greenhouse gases (4). They are also found in the human and animal digestive tract, where some aid in food digestion and nutrient delivery to the host, while others may be involved in pathogenicity or providing resistance to antimicrobials (5, 6). Anaerobes are also essential for biotechnological processes, such as anaerobic digestion, biomanufacturing commodity chemicals, and fuels or bioremediation (7–9). Therefore, anaerobes are of significant interest across environmental, and clinical microbiology disciplines.

In the last decades, there has been great progress on elucidating microbial diversity within anoxic environments, mainly due to the introduction of DNA-based sequencing methods. Yet, microbial cultivation and physiological studies remain critical to understanding the metabolism of anaerobic microorganisms and their response to biotic and abiotic factors. Thus, the development and integration of novel culturing methods and microscopic techniques under anoxic conditions, mimicking the conditions in which anaerobic microorganisms live, are essential.

Anaerobes are inherently challenging to study due to their slow growth and sensitivity to oxygen. Besides, method development for anaerobic cultivation has been slow or stagnant. The methods of Hungate (1950, 1969) (10, 11) and Balch (1976) (12) remain the cornerstones of current lab techniques despite being laborious and time-consuming. An overview of anoxic cultivation techniques has been extensively reviewed elsewhere (4, 13). One of the drawbacks of anoxic culturing is the reliance, in most cases, on batch growth conditions with a typical shift from high to depleted nutrient conditions, which are not reflective of true environmental conditions. Anoxic continuous culture or chemostat cultivation is possible although tedious and dependent on expensive equipment and lab infrastructure to keep anoxic conditions, e.g., an anaerobic glovebox or glove bag, also known and sold as anaerobic chambers (14). However, all traditional anoxic culturing approaches fail to provide insight into the individual cell responses, cell spatial organization, and cell-cell interactions (such as chemotaxis, changes in individual cell growth rates, and physiological cell changes) of anaerobic microorganisms. Research has, therefore, come to depend on time-lapse culture-independent omics and cell-destructive microscopy approaches to provide snapshots into these missing links. These snapshots give hints regarding cell-to-cell interactions, but ultimately, experimental validation is needed to find causal relationships. We, therefore, lack a mechanistic understanding of how key anaerobic taxa interact with their environment and each other to ultimately influence biotechnology, environmental or host ecosystems at the macroscale level (15, 16).

**Fig 1:**
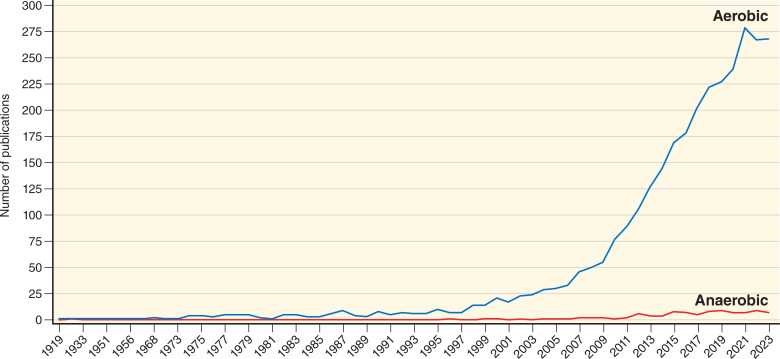
Results of a search from PubMed showing the results for searches for “Aerobic” single-cell bacterial studies (“Bacteria” AND “Single-cell”—3,086 papers) and “Anaerobic” single-cell studies (“Bacteria” AND “Single-cell” AND “Anaerobic”—98 papers).

To achieve this mechanistic understanding, we need to radically redesign how we conduct our experiments. Previous studies have proposed, for example, sophisticated non-destructive single-cell physiological experimentation, which they termed “next-generation physiology” (17). This approach uses a combination of culturing, phenotyping, sorting, and processing of cells of interest to yield insight into how individual cells respond to environmental conditions (or other tested parameters). However, to apply such an approach to anaerobes while maintaining non-destructive experimentation (to maintain viable cells) is still incredibly challenging. In contrast, aerobic culturing has experienced a “cultural renaissance” (18), where innovative cultivation efforts and single-cell applications, such as high-throughput lab-on-chip and microscopy methods, combined with genetic engineering and genomics are quickly transforming our understanding of the dynamic and subtle interactions between members of microbial consortia in complex environments [e.g. soils (15)], or the gut (19). Single-cell technologies involve experimenting with or visualizing single cells from microbial populations. A search on PubMed (National Library of Medicine, NIH) shows that manuscripts using aerobic single-cell applications totaled over 3,000 up to December 2023, whereas anaerobic single-cell applications yielded fewer than 100 publications during the same time period (Fig. 1).

**Fig 2:**
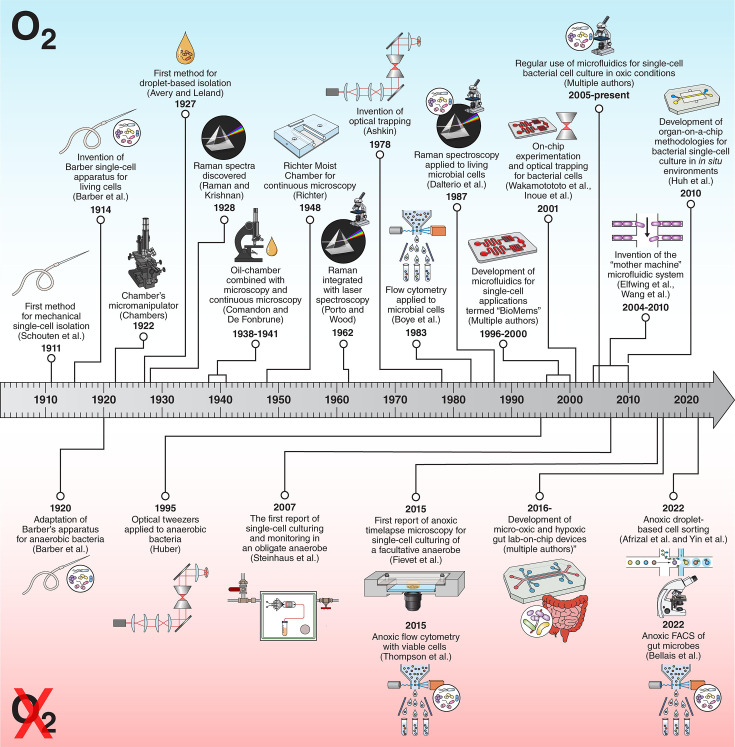
Timeline showing the developments in aerobic microbiology (shown on top) parallel to the developments in anaerobic microbiology (shown on the bottom, represented with crossed out O_2_) in the context of single-cell analysis images and instrumental advances. The full list of references is provided in File S1.

Figure 2 illustrates a timeline parallel of developments in aerobic and anaerobic single-cell microbiological studies. The very first bacterial single-cell isolators were invented as far back as 1911 (20), such as the Barber’s single-cell isolator (21) and the Chambers micro-manipulator (22). Barber adopted the single-cell isolator for anoxic conditions in 1920 (23). From this point on, several developments were made concerning aerobic single-cell culture, such as droplet isolation (24), and oil-chamber and continuous microscopy (25, 26). Although these techniques were first adapted for anaerobes in 1941 (27), they were not widely implemented. Only recently have droplet-based single cell-sorting techniques been revived for anaerobes (28, 29).

The next breakthrough in aerobic single-cell applications occurred with the invention of optical trapping of particles using lasers by Ashkin in 1978 (30), which was later applied to bacteria in 1987 (31). It took almost a decade for this methodology to be adapted to anaerobes by Huber et al. in 1995 (32). From the late 1990s, (aerobic) single-cell applications experienced exponential growth with the development of advanced microscopy facilities for timelapse (e.g., automated stages, autofocus capability, and digital imaging) and the innovation of BioMEMs (biomedical or biological microelectromechanical systems) or lab-on-chip applications, initially for mammalian cell-culture and other biological applications (33–35). These were rapidly applied to bacterial single cells for their culture and isolation (36, 37). Microfluidics for (aerobic) bacterial single-cell applications became more common from 2005 onward (38–40) and the incremental development of the mother machine device to monitor individual cell growth rates (41, 42). The next major advancement has been the development of highly sophisticated *in situ* environments for single-cell analysis such as the “organ-on-a-chip” (43). Adoption of lab-on-chip devices to anoxic single-cell applications has been slow and only carried out by a handful of researchers (Fig. 2), for example, the use of anoxic single-cell pure-culture in 2007 (44) and again in 2015 (45). However, developments in gut-on-a-chip applications have been substantial (19, 46).

The slow adoption of aerobic single-cell imaging techniques to anoxic applications is likely due to the multidisciplinary skillset needed to develop these experimental conditions (for example, nanofabrication and fluid handling) and the technical challenges of maintaining anoxic conditions. Handling anaerobic microorganisms presents unique challenges due to the requirement for low-oxygen or oxygen-free environments to maintain their viability and physiological properties. Some challenges associated with using single-cell imaging methods with anaerobic species include sample handling, imaging setup, live-imaging, fluorescent probes, and cell viability. Therefore, specialized equipment and techniques are needed, for instance, closed imaging systems to secure anoxic conditions, which often require customized setups, or new labeling strategies to visualize cells and their cellular processes.

In this mini-review, we provide a comprehensive overview of single-cell applications in anoxic conditions involving live-cell conditions. We present the key advances, from single-cell imaging, microfluidic experimentation, and cell sorting. We further highlight the challenges and limitations of anoxic single-cell applications and pose exciting future research opportunities.

## SINGLE-CELL IMAGING IN ANOXIC CONDITIONS

As an integral part of modern cell biology, single-cell imaging encompasses the exploration of living cells through fluorescence microscopy. It enables researchers to visualize biological processes and track and quantify molecules. Imaging of living anaerobic cells has two major requirements: first, labeling the cells while keeping them anoxic and viable and second, maintaining anoxic conditions during imaging. Visualization based on fluorescence proteins (FP), dyes, and other fluorophores is widely used under oxic conditions. Using genetically encoded FPs to specifically label molecules in living cells also circumvents the challenge of cell permeability of externally added dyes or molecules, e.g., antibodies, being then one of the most widely used tools in fluorescence microscopy. A broad range of FPs with different properties, such as color, pH, and temperature stability (47–50), photo-conversion (51), photo-activation (52, 53), or split variants for protein-protein interactions (54), are readily available. However, most of these conventional FPs, such as the blue-green fluorescent protein (GFP) (55) or red mCherry (56), are dependent on the presence of O_2_ to develop a fluorescence signal (57, 58). In fact, GFP has been used as a reporter for hypoxia at O_2_ concentrations of at least 0.1%, while conditions below 0.02% O_2_ lead to a significant loss of fluorescence signal (59), clearly showing the limitation of GFP in oxygen-limited experiments. FPs like GFP or mCherry have been used as reporter proteins for anaerobic organisms; however, O_2_ was added at some point to the culture, during fixation or imaging to obtain the fluorescence signal (45, 60). It is expected that the necessary oxygen concentrations to obtain a fluorescence signal would lead to oxygen-stress in the cells during live-cell imaging with consequences in growth behavior or loss of viability (45), which greatly limits the application of FPs for live-cell imaging of anaerobic microorganisms.

### Imaging methods based on genetically encoded labeling

In the past years, alternative fluorescence labeling methods have been established to compensate for the lack of oxygen-independent FPs (61–63). One of the first FPs demonstrated to function as fluorescence reporter genes in live anaerobic bacteria was Flavin mononucleotide (FMN)-based fluorescence proteins (FbFPs) (62). They are based on the bacterial blue-light sensitive and photoactive light-oxygen-voltage domain (LOV). Different variants of FbFPs such as iLOV, evoglow, and miniSOG are known to be functional as fluorescent markers in gut bacteria under different biological conditions (64–68) but also in eukaryotic cells under anoxic conditions (63, 69). The characteristics and applications of FbFPs have been reviewed before (70). The advantage of FbFPs is their small size and the availability of FMN in most cells. However, the fluorescence signal emitted by FbFPs remains dim in comparison to enhanced GFP (eGFP). In addition, the signal is in the blue spectral range and, therefore, overlaps with intrinsic cellular fluorescence making the differentiation of proteins labeled with FbFPs challenging. This can especially be a disadvantage when studying methanogenic archaea and some bacteria which possess a strong green blue autofluorescence attributed to the methanogenic redox cofactor F_420_ or other fluorescent molecules present in cells (71, 72). Several studies have been performed to generate FbFPs with shifted spectra by exchanging amino acids forming a stable FPs with moderate success (73).

Bioorthogonal labeling approaches for live imaging have gained traction in recent years (74, 75). There are a variety of bioorthogonal labeling methodologies. In this section, we will discuss those which require *a priori* knowledge of the genetic system and have been tested in anaerobic organisms. For example, self-labeling protein tags, such as HaloTag (Promega) (76), SNAP-tag (77), or CLIP-tag (78) (New England Biolabs), enable the attachment of ligands that provide high fluorescence signals and are more photostable than FPs. These tags are highly specific to their ligand forming an irreversible protein-ligand bond. Ligand libraries with a broad range of spectral behaviors are available. The HaloTag system has been shown to be functional for anoxic single-molecule imaging of *Bacteroides thetaiotaomicron* (79, 80). Furthermore, SNAP-tag, CLIP-tag, and HaloTag have been demonstrated to be suitable anoxic fluorescence reporters in some *Clostridium* species and *Porphyromonas gingivalis* (81–83). The organic ligands, however, can be cytotoxic, are often poorly cell permeable, and can require excessive washing to avoid background fluorescence. In anoxic applications, extensive wash steps are opportunities for oxygen intrusion. An alternative innovative approach is the use of fluorogenic proteins, including the bilin-binding UnaG, infrared FP IFP2.0, and near infra-red FPs, which rely on protein-ligand complex formation for fluorescence signal generation, thus mitigating the need for washing steps. UnaG has similar spectral characteristics as GFP and has been demonstrated to be functional under anoxic conditions during two-color imaging with IFP2.0 in *Bacteroides* species and in mammalian cells (84–87). IFP2.0 emits fluorescence in the far-red spectrum. The emergence of the fluorescence-activating absorption-shifting tag (FAST) has further expanded the anoxic imaging toolkit, with successful applications in diverse bacteria and archaea (88–92), offering reversible, quantitative, and bright fluorescence upon ligand binding. Nevertheless, challenges persist. Oxygen-independent fluorescence labels often require exogenous ligands, introducing limitations due to cellular permeability. So far, most fluorescence labels are either used for protein localization or tracking and gene expression analysis. However, the development of split variants of SNAP-, SNAP/CLIP-, and FAST-tag for protein-protein interactions might lead to future studies of cellular processes in anaerobic organisms (93–95). Besides FPs and self-labeling protein tags, FlAsH, a biarsenical derivative of the dye fluorescein-5-isothiocyanate (FITC), has been used in *B. thetaiotaomicron* for live single-molecule fluorescence imaging of an outer membrane protein under anoxic conditions (80). FlAsH covalently binds to a tetracysteine motif introduced into the protein sequence.

**Fig 3:**
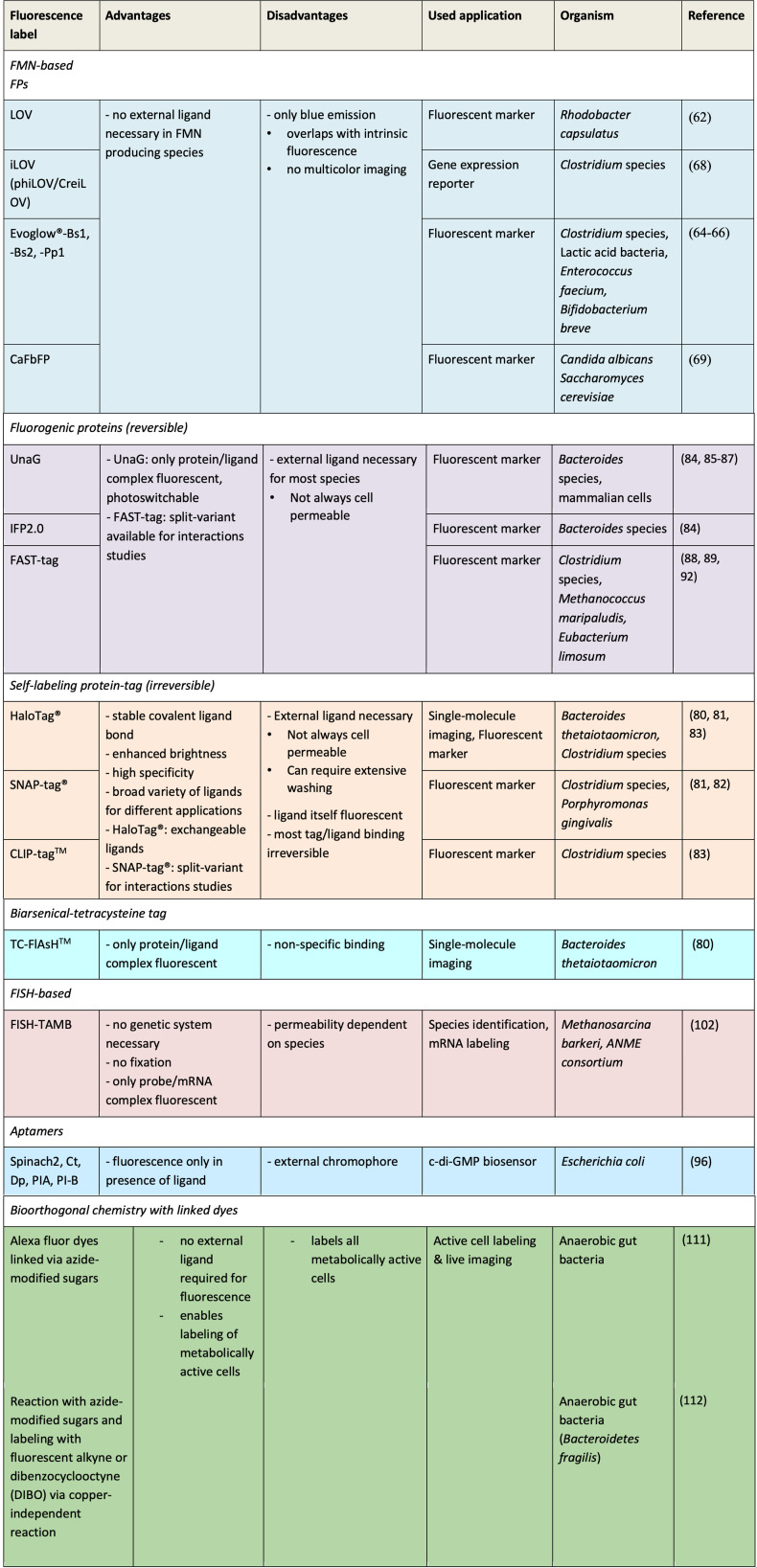
Overview of fluorescence labeling methods used under anoxic conditions.

RNA-based fluorescent biosensors like Spinach2 have been shown to detect c-di-GMP in *E. coli* under anoxic conditions (96). The Spinach2 aptamer is a short single-stranded RNA molecule which binds an external chromophore. Fused to a second detection aptamer, the fluorescence signal is dependent on the presence of a ligand. It provides high fluorescence, fast activation, and specific binding to study c-di-GMP signaling in live bacteria under oxygen-free conditions. In addition, Wang et al. (96) have proposed that related aptamer-dye pairs could also be used for other small molecules or proteins and for imaging of gene expression levels. Another oxygen-independent fluorescence labeling is expanding the genetic code by incorporating unnatural amino acids (UAAs) into proteins. This method enables fluorescence live-cell imaging and bypasses the disadvantage of bulky fluorescence labels and, therefore, compatible with cell viability. Genetically encoded UAAs provide target-specific labeling (97). With a minimal distance between probe and target, UAAs have been successfully used for super-resolution microscopy. Several fluorescent amino acids to be incorporated at amber codons are available (98, 99). An overview of anoxic fluorescence microscopy labeling methods is shown in Fig. 3.

### Alternative labeling strategies for microbial species without genetic systems

The FP overviewed above rely on the use of expression vectors, and thus, they require the availability of genetic systems and expression vectors for the target microorganism. Unfortunately, genetic systems are only available for a limited number of microbial species. This excludes the use of expression vector-coded FPs for yet-uncultured microorganisms or those with a non-yet established genetic system. To circumvent this problem, an alternative is the use of fluorescence labeling strategies with non-genetically encoded labels, like fluorescence *in situ* hybridization (FISH) methods.

Conventional FISH-based methods are typically destructive for anaerobes, requiring long and complicated protocols (up to 30 h) including fixation, permeabilization, and washing steps. Fixation-free FISH methods omit the fixation step but still need long incubation times for hybridization and washing (100, 101). These steps can compromise cell integrity, leading to a loss of cell viability. While these methods can be used for downstream applications like proteomics or metagenomics, a shorter protocol reducing oxygen exposure and free of chemicals that could damage the cells would be preferred for continuous imaging of viable living cells. FISH of transcript-annealing molecular beacon (FISH-TAMB) requires only a short incubation time (which can be performed in an anaerobic chamber), and it is fixation-free, both minimizing the risk of oxygen contamination and of cell damage considerably (102). FISH-TAMB has been used for imaging of the strict anaerobic archaeon *Methanosarcina barkeri* and anaerobic methanotrophic archaea, leading to the detection of non-culturable taxa, active gene expression, and different transcript expression levels. Harris et al. have shown that FISH-TAMB-treated cells show little impact on cell viability (102). As it is a quite new method, not many studies using FISH-TAMB are published, and it likely needs further improvements to be used for other species.

Alternatives to FISH-TAMB could be, for example, small cell permeant fluorescence dyes, e.g., MitoTracker, CellMask, or SYBRSafe dyes (Invitrogen), which are independent of both molecular oxygen and of an available genetic system. These stains have been shown to be non-cytotoxic and lead to efficient fluorescence staining under different extreme physiological conditions, e.g., high-salt, high-temperature, and low pH (103, 104). However, the dye permeability and functionality are strongly dependent on the investigated organism and its physiological characteristics. Nanobodies (tiny, recombinantly produced domains of heavy-chain only antibodies) and chromobodies (small intracellular functional antibody genetically fused to an FP) represent further options to broaden the spectrum of available live-cell imaging methods for anaerobic organisms (105) as their functionality is independent of oxygen. However, their applicability in anaerobic microorganisms has yet to be demonstrated.

Bioorthogonal fluorescence labeling approaches that do not require prior knowledge of the cell’s genetic system are an alternative solution for studying *in situ* interactions in anaerobic microbial communities (106). These methods enable protein tagging, cell labeling, and tracking physiological activity in microbial communities in a broad range of conditions by using labels that minimally interfere with the biology of the cell (107). In terms of anaerobic applications, methods like bioorthogonal noncanonical amino acid tagging (BONCAT) have been applied to label the translationally active members of microbial communities. BONCAT-labeled cells can be further visualized and identified using FISH as shown in an anaerobic methane-oxidizing (ANME) community (108) and various bacterial and archaeal isolates (109). However, the FISH step in these cases requires genetic information to design specific probes. Alternatively, BONCAT can be combined with click chemistry for imaging using oxygen-independent fluorophores, as shown in studies where BONCAT with TAMRA-alkyne was used to investigate protein expression during anaerobic survival in *Pseudomonas aeruginosa* (110). A limitation of these techniques, including the previously mentioned methodologies for fluorescence microscopy, is the limited number of applications under anoxic conditions in live-imaging experiments and, therefore, limited information on cell viability (Fig. 3). Thus, it remains often unclear how the cells are affected in their viability by factors such as label and reagent toxicity, and efficiency of label uptake. Live-imaging conducted in the gut environment suggests that copper-free BONCAT reactions improve cell viability as tested in the anaerobic bacterium *Bacteroidetes fragilis* (111, 112). Increased use of bioorthogonal methods in anoxic conditions will improve the methodology and expand the toolbox that can be used to study anaerobes and their role in communities.

### Imaging methods using microfluidic experimentation

Microfluidic (or lab-on-chip) devices can confine cells into a controlled space, allowing their visualization and tracking using high-resolution microscopy. Chemical conditions in these devices can be tightly controlled to mimic *in situ* conditions, and depending on the device design, the placement of microbial cells can also be configured. Single-cell microfluidic platforms can show heterogeneity in individual cell growth rates and other cell responses that would otherwise be masked by batch culture (113). For a review of single-cell microbial cultivation using microfluidics, see Anngraini et al. (114). Despite this field experiencing exponential growth from the early 2000s, applications in anoxic conditions remain relatively few. We will discuss the key advances in single-cell imaging using microfluidics under anoxic conditions below.

**Fig 4:**
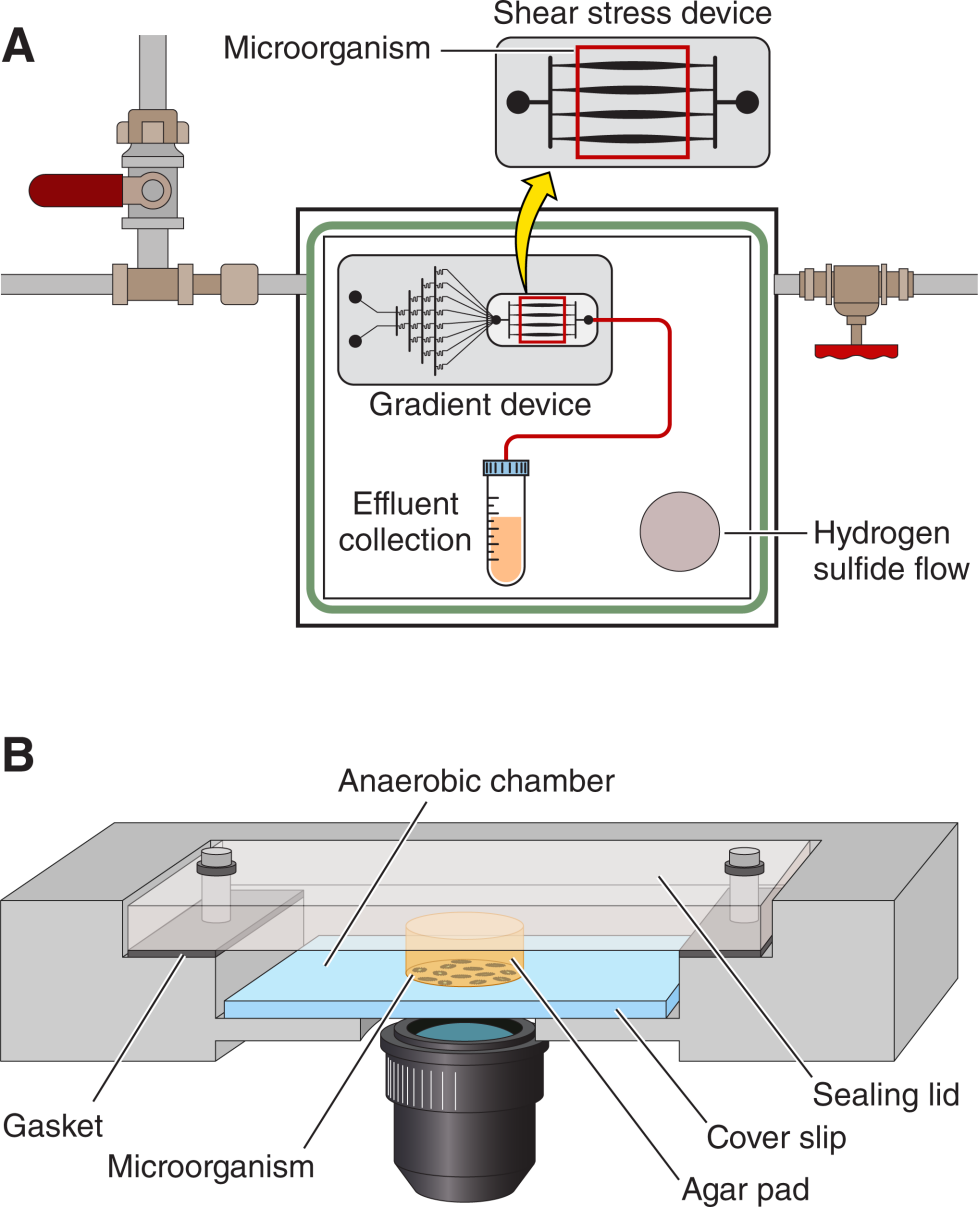
Schematic of two anoxic single-cell applications using microfluidic experimentation. (**A**) shows the system developed by Steinhaus et al. (44) which uses microfluidic devices encased in an anoxic environment sparged with N_2_ gas. The device was placed in an anaerobic glovebox and imaged on a microscope once per day. (**B**) shows the system employed by Fievet et al. (45) and adapted from Ducret et al. (115) which employs a simple coverslip and agar pad which was closed via plates and gaskets and continuously monitored on a high-throughput microscope.

One of the first instances of anoxic (anaerobic), single-cell imaging using microfluidics was performed by Steinhaus et al. (44) using the methanogen *Methanosaeta concilli*, arguably one of the strictest anaerobes known (44). This sophisticated system contained a sealed portable chamber containing either a shear flow or concentration gradient microfluidic device (Fig. 4A). Hydrogen sulfide was used to scavenge oxygen from the chamber. Additionally, the system was placed in an anaerobic glovebox and was removed once per day for imaging purposes. During imaging, the system was sparged with N_2_ to minimize oxygen intrusion. Using this setup, the authors determined the optimum shear rates for maximum biomass production, as well the pH and ammonium concentrations required for optimal growth of *M. concilli*. It was over a decade later that single-cell imaging in anoxic conditions was again applied to an environmental organism (115). In this application, *Desulfovibrio vulgaris* was cultured in a microfluidic device combining a cover slip flow chamber with an agar pad, sealed within a gasket chamber [(45); Figure 4B]. The device was continuously monitored under the microscope. Anoxic conditions, however, were not stringent as the authors tested the response of *D. vulgaris* to oxygen stress. Real-time visualization with high-resolution microscopy was conducted, with cells visualized via fluorescence using GFP labeling (requiring oxygen exposure). The study provided valuable insight into cell division in *D. vulgaris* and showed that cell division was halted by oxygen exposure. However, the agar pad methodology used in the study has disadvantages such as the squeezing of cells, challenges in tracking cells, and difficulty in maintaining a homogenous environment during experimentation (116).

In clinical research, there has been a surge in organ-on-chip devices to study host systems. One such area is the interface between host cells and anaerobic microbial populations (for example, the intestinal system), which has been extensively reviewed elsewhere (117–119). A review by Bossink et al. (120) paid particular attention to anoxic systems, covering progress, and considerations such as oxygen sensing and device fabrication (120). Some examples of anoxic applications include Shah et al. (19), Greenhalgh et al. (121), Jalili-Firoozinezhad et al. (46), Wang et al. (122), and Shin et al. (123), achieving variable oxygen concentrations from 0.1% to 0.9% oxygen. These authors all used some variation of a microfluidic device to monitor the interaction between an endothelial cell layer and anaerobic bacteria. Most included oxygen-sensing capacity in the devices. However, given the reported variation in sensor capacity, oxygen monitoring, and reported concentrations (often in percentages rather than in parts per million, ppm), therefore, these studies may be considered to be performed in micro-oxic or hypoxic conditions). While these examples highlight the technical challenge of hypoxic to micro-oxic cultivation in complex *in situ* environments with the capacity for single-cell imaging, the focus was geared toward culturing and other downstream applications, and thus, they lack insight into the physiology and activity of individual anaerobic microorganisms.

Moreover, the complex intestinal microfluidic systems described above tend to be used to culture facultative anaerobes and aerobes in layers and have the advantage that the system itself can become oxygen scavenging as oxygen-tolerant bacteria use oxygen in the system thereby increasing the anoxic conditions over time (123, 124). Interestingly, a study by Grant et al. (125) further advanced the organ-on-chip and assessed various means of oxygen control (respiration of aerobic cells, microfluidic device coatings, and fluid flow) to establish hypoxia (125). Nonetheless, it is anticipated that pure culture strict anoxic single-cell studies will be more challenging. However, these advances showcase the potential to adapt such technologies to the field of environmental anaerobic microbiology to reveal, for example, phenotypic heterogeneity (e.g., individual cell growth rates) in biotechnologically relevant organisms, the mechanisms underpinning syntrophic interactions, and, indeed, cellular mechanisms (e.g., cell division) in anaerobic microbes.

## CELL SORTING UNDER ANOXIC CONDITIONS

**Fig 5:**
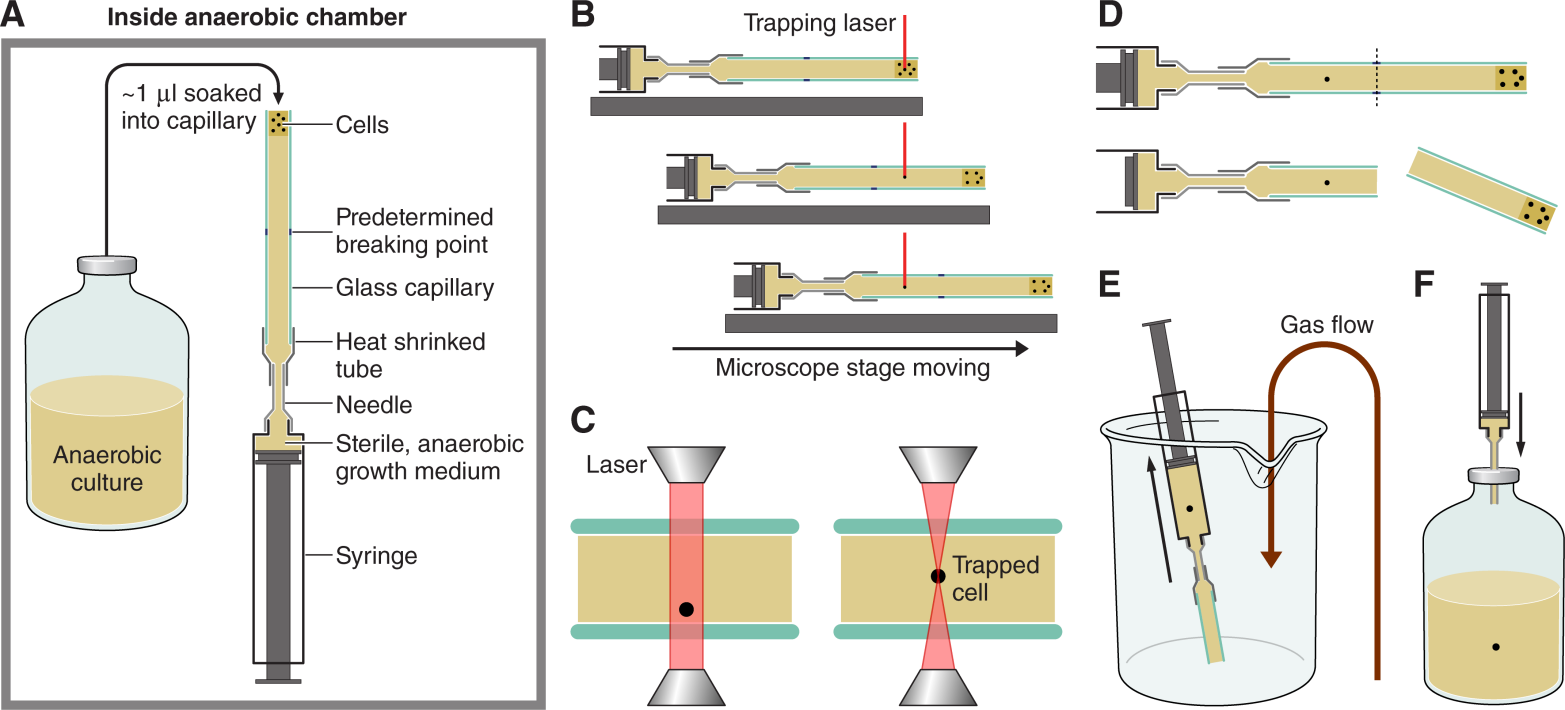
Isolation of a single cell via Optical Tweezer. To avoid exposure to oxygen, an anaerobic glovebox should be located next to the microscope. (**A**) A 1-mL syringe with a needle is connected via a tube to a rectangle glass capillary (inside 0.1 × 1 mm, length 10 cm) with a predetermined breaking point. The connection tube is heat-shrinked. The needle and capillary are filled with sterile, anaerobic growth medium up to 90% of the capillary volume. About 1 µL of an anaerobic culture is soaked into the open end of the capillary. (**B**) The capillary with the syringe is fixed on a microscope stage of an inverted microscope (Axiovert IM35, Zeiss, Oberkochen, Germany). For imaging, an oil immersion objective (100/1.3) and a Nd-YAG laser are used (wavelength 1,064 nm; maximum power 1 W, ADLAS, Lübeck, Germany). The cell suspension is imaged under 1,000×-fold magnification. Cell separation happens by moving the microscope stage and (**C**) optically trapping a single cell in the laser beam. (**D**) After separation, the capillary is taken out of the microscope and cut into two pieces at a predetermined breaking point. (**D**) Immediately, the syringe is put into a beaker filled with anoxic gas, and the cell is pulled “anoxically” into the syringe and (**E**) flushed into fresh anoxic growth medium for incubation [modified after reference (32)].

The first attempts to perform cell isolation for anaerobes were done over a hundred years ago using an instrument specifically designed for anaerobic single-cell isolation (23). However, despite several successful isolation attempts using this method, it was considered challenging and was even described to be “wasteful of time, material, eyesight and nervous energy” (126). Currently, single-cell sorting of anaerobes is no less challenging, but with the development of automated systems and modernization of lab infrastructure, new attempts can be made to separate microbes from their communities (127, 128). Thompson et al. (129) used a cell sorter equipped with a sealed chamber fluxed with N_2_ to perform the selection of the cells and the sorting, and an anaerobic glovebox around the stage where the cells were deposited after sorting, enabling sorting of cells in anaerobic fashion. This enabled the isolation of individual members of a co-culture community and allowed the assessment of the cell viability of differently evolved cell lines, showing the feasibility of this approach (129). Similarly, using fluorescence-activated cells via antibodies, Bellais and co-workers enabled cell sorting using flow-cytometry equipped with an anaerobic glovebox and obtained viable strict anaerobic pure cultures from fecal samples (130). Droplet-based cell sorting could similarly be used to obtain a large spectrum of viable anaerobic isolates without the use of labels (28, 29). This droplet-based approach was performed in an anaerobic glovebox and required a shorter experimental timeframe compared to the traditional plating approach and resulted in fast isolation of viable anaerobic pure cultures from fecal samples. A different approach to isolate individual cells from complex communities is via optical tweezers, where a strong light bundle is used to trap individual cells and move them to a separate environment. This method was applied to achieve viable pure cultures of anaerobic archaea (32). Briefly, the target cell is identified and fixed via a laser beam. The microscope stage is used to maneuver a single cell from the mixed sample inside a glass capillary connected to a micro-syringe. The glass capillary is then cut to separate the single cell from the mixed cells at the end of the capillary. The syringe is used to transfer the cell directly and swiftly into fresh anaerobic culture medium, enabling subsequent cultivation (Fig. 5). With a similar approach, this method has also been applied to co-cultures of the anaerobic archaea *Nanoarchaeum equitans* and *Ignicoccus hospitalis*, to study their interaction (131). While the optical tweezer approach in these studies is not described as being performed in anoxic fashion (e.g., in an anaerobic glovebox), still viable cells of anaerobic archaea could be obtained. It remains a question if more strict anaerobes, such as methanogenic archaea, would survive such a procedure.

The methods described above all rely on some form of selection, via either labeling or morphological identification (e.g., cell shape), and therefore, rely on prior knowledge about the target. In order to label and sort relevant microbes from undefined (and often poorly understood) anaerobic communities, bioorthogonal chemistry approaches as described previously are an effective alternative (106). BONCAT can be followed up by FISH or fluorescence-activated cell sorting (FACS) and sequencing, allowing the user to sort and identify the active members of the community, as demonstrated in an anaerobic methane-oxidizing (ANME) community (108), anammox (132), and wastewater communities (133). If the cell sorting technique requires the anaerobes to remain alive, all reactions should be able to proceed in the absence of oxygen and, thus, also require fluorophores that work in such conditions. Further development of these bioorthogonal chemistry approaches could open up research opportunities related to fundamental physiology, spatial organization, host-microbe interactions, and growth rate assessments of strict anaerobic communities.

**Fig 6:**
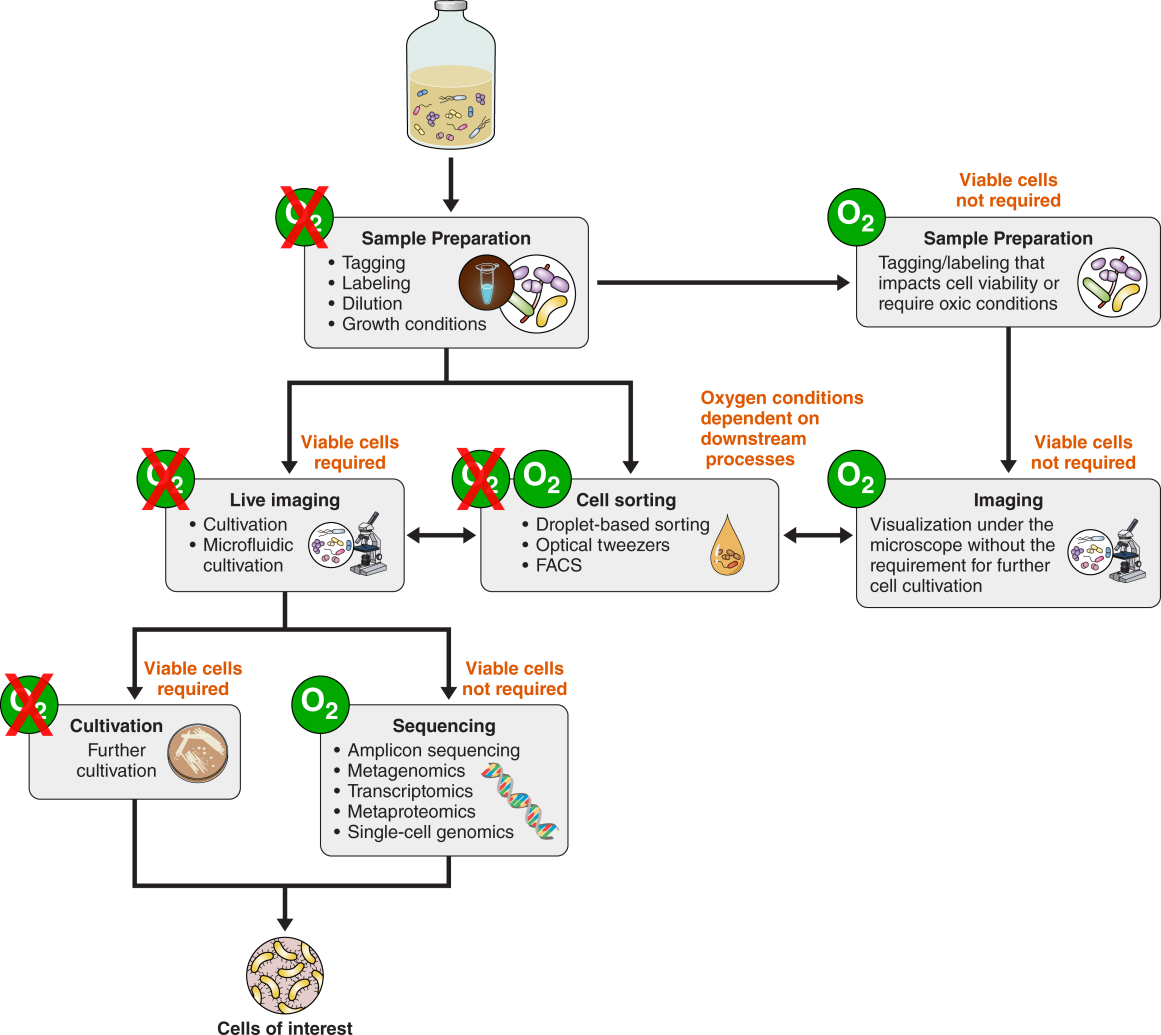
Overview of a sample experimental anoxic single-cell cultivation workflow and downstream processes, outlining where anoxic conditions may be required. Oxic/aerobic is represented with O_2_, and anoxic/anaerobic as crossed out O_2_. Note: Raman spectroscopy could be considered under the “imaging” or “live-imaging” heading depending on the approach used and the requirement for viable cells.

Cell-sorting applications open a world of possibilities for anaerobes when integrated with downstream pipelines (Fig. 6). For example, single-cell RNA sequencing and single-cell genomics or strain-resolved genomics, while still technically challenging, can already be applied to some microbes (134). This gives insights into the transcripts, function, and taxonomy of individual cells. These types of methods do not require maintenance of anoxic conditions after the cell harvesting procedure, allowing it to follow the original protocols. Other applications, however, like live-cell-imaging or follow-up cultivation, do require maintenance of anoxic conditions after sorting; then the further downstream pipelines need to be specifically designed to maintain anoxic conditions (Fig. 6).

## FUTURE RESEARCH OPPORTUNITIES

Techniques showing promise for future anoxic single-cell studies are highlighted below as well as the technical challenges to overcome and examples of lessons learned from aerobic studies.

### Parallels to aerobic cultivation

**Fig 7:**
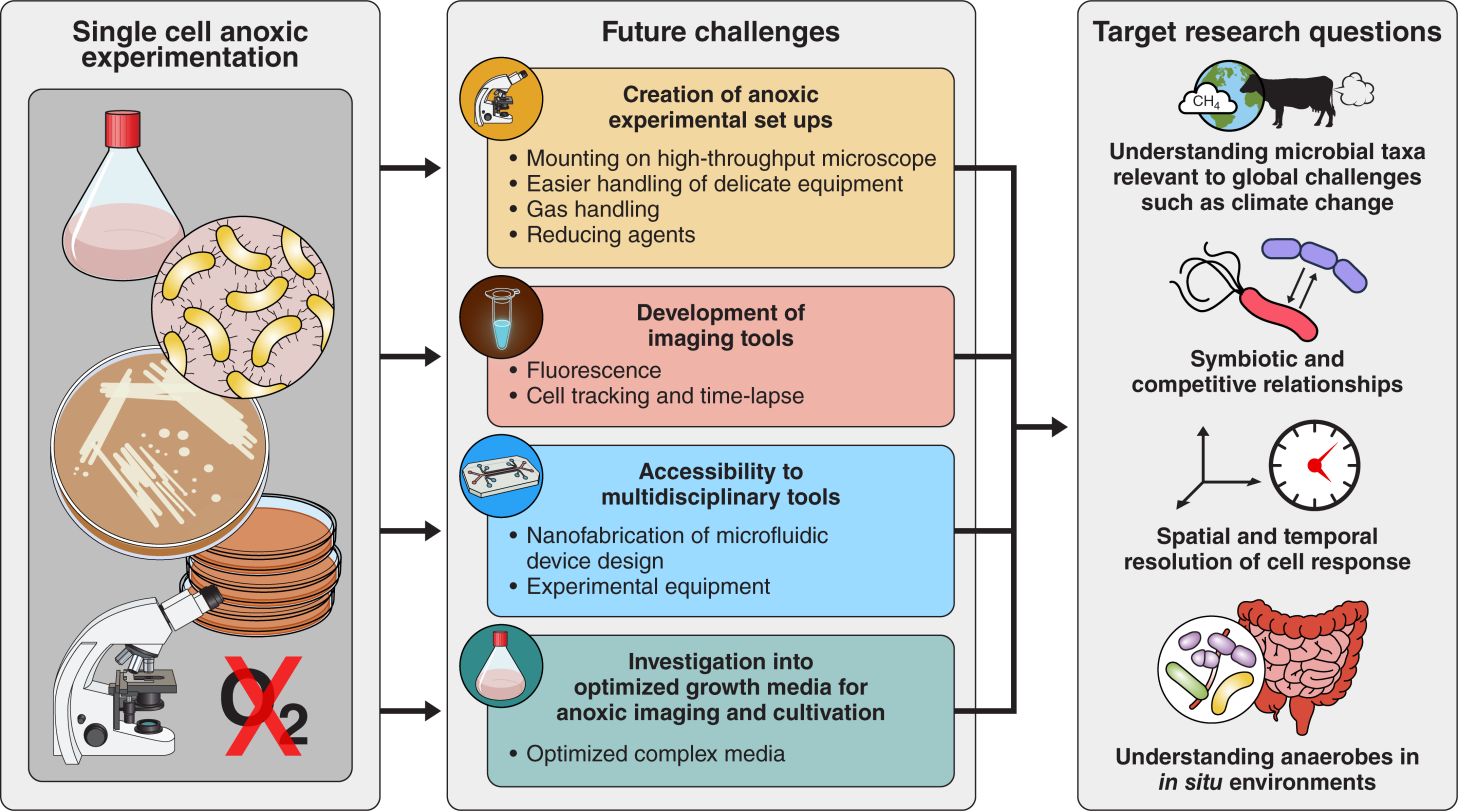
Overview summary describing the future challenges to be solved and potential research questions advances that single-cell anoxic applications could solve. Anoxic/anaerobic is represented with crossed-out O_2_.

In this review, we have focused on the advancements made in anoxic single-cell studies. Many of these studies focus on method development, and this is warranted given the lack of tools available for anoxic single-cell cultivation. To highlight the value in these method developments, we will briefly describe interesting scientific discoveries that have been derived from aerobic single-cell cultivation studies. Microfluidics cell sorting and isolation have been used to overcome the problem of the uncultured majority, thus, identifying novel species (135). Samlali et al. (136) used droplet-based cell sorting to sort fungal cultures with a particular trait of interest—cell-wall-degrading enzymes to rapidly develop a population with the maximum desired activity (136). Microfluidics cultivation has also shed light on the phenotypic heterogeneity of clonal bacterial populations (137). Cell growth rates and population evolutionary history can also be determined using the Mother machine, which consists of trapping the mother or parent cell in a microchannel’s dead-ends of a microfluidic device and letting the population grow (42, 138, 139). Microfluidics have also been used to elucidate microbial cross-feeding, cellular communication, and response to resource variations (e.g., (15). Similar advancements could be made in the field of anaerobic microbiology if these innovative methods were adapted accordingly (Fig. 7).

### Raman spectroscopy

One technique which shows a great deal of promise for future anoxic live microbial single-cell applications is Raman spectroscopy. Raman has traditionally been used in chemistry, material science, and the pharmaceutical industry but, in the past 15 years, has seen increased use in varying subfields of microbiology (140). In Raman spectroscopy, a laser light source is applied to a sample and the scattering of this light can be used to determine information on the molecular bonds and chemical structure of the sample. It is a non-destructive and rapid technique that can provide metabolic and physiological information from single cells. Lee et al. (2021a) provide an extensive overview of the potential of different Raman methodologies for microbial cells (140). For living cells, the technique can be used to analyze communities *in situ* in liquid culture. Regarding anaerobic microorganisms, these could be analyzed *in situ* as long as anoxic conditions are met, and potentially, they would be viable after the analysis to proceed with anoxic downstream analyses (Fig. 6).

Specifically, in the application of Raman microspectroscopy, an integrated microscope can be used to select the area of interest in the sample. The sample can be held within a microfluidic device for highly controlled fluid flow (141, 142). Labeling approaches (see above sections on imaging labeling with and without a known genetic system) are advised as the application of Raman techniques to mixed and even pure cultures would generate a lot of noise due to variations in cellular composition and environmental conditions in the liquid medium (143, 144). Raman microspectroscopy has been also used in combination with stable isotope probing (141, 142) to label cells with the desired metabolic phenotypes of interest. FISH has also been applied together with Raman microspectroscopy to better pinpoint the diversity of the targeted microorganism(s) (145). Note that to maintain viable labeled cells during Raman, non-toxic labeling approaches may be more appropriate here (e.g., genetic labeling, FAST-tag, FISH-TAMB, or other approaches as outlined in Fig. 3). However, these labeling approaches are currently untested in combination with Raman microspectroscopy. Cell sorting via optical tweezers can then be used to sort the cells for downstream applications (142). The study by Lee et al. (142) highlights the development of a fully automated optofluidic platform for Raman-activated microbial cell sorting (RACS), and the authors provide a detailed protocol (146). To adapt these procedures to anoxic cultivation experiments would require placing the entire experimental set-up inside an anaerobic glovebox, which may be challenging due to the space requirement and additional risk assessments required for working with lasers and hydrogen mixed gas. The more feasible option would be to create an anoxic cultivation platform that could be embedded on the microscope or around the sample stage. For example, an oxic/hypoxic microfluidic system for mammalian muscle cell culture has been integrated with Raman spectroscopy previously although the exact oxygen concentrations were not reported (147). Other considerations would include anoxic medium development compatible with this approach as Raman requires a non-photoluminescent media not masking cellular signals. In microfluidic systems, additional considerations include the device material and gas flow which may interfere with the Raman signal (142, 147).

### Technical challenges for future anoxic single-cell applications

**Fig 8:**
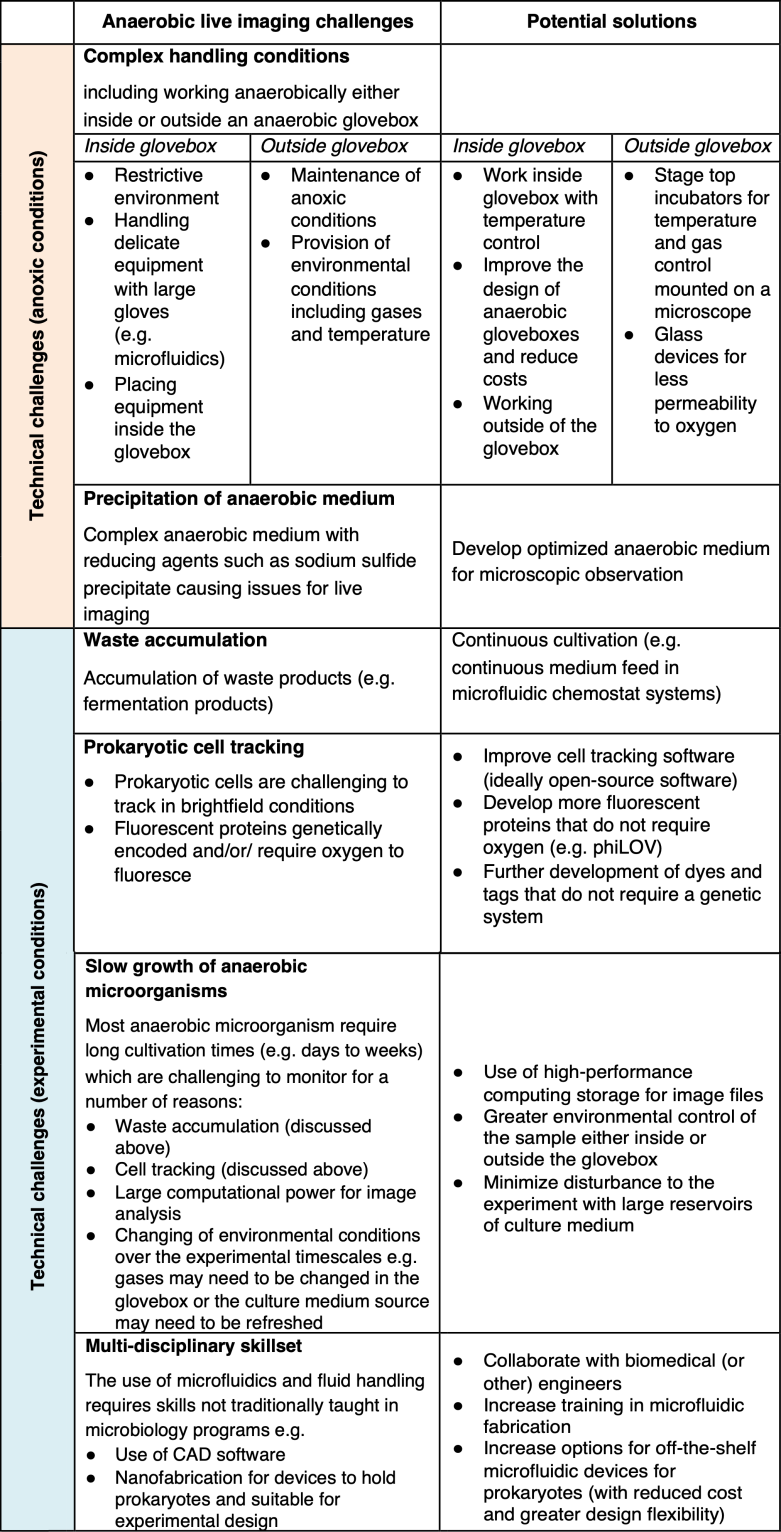
Overview of the challenges for anoxic live-imaging and suggested solutions.

While, in recent years, significant developments have taken place, anoxic single-cell techniques remain underdeveloped compared to oxic methods. The requirement for the absence of oxygen results in physical challenges of equipment placement in anaerobic gloveboxes, resulting in more complex handling conditions and more expensive set-ups. For techniques operated on a relatively short timescale (e.g., cell sorting, short term-imaging), anoxic requirements are less strict and can be viable as long as oxygen exposure remains limited. Significant challenges arise, however, when it comes to prolonged experiments looking at population dynamics or extended growth curves, which are the common questions tackled with live-cell imaging methods. Due to the relatively slow growth of anaerobes, they suffer from environmental changes (e.g., substrate depletion, waste accumulation) that need to be controlled anoxically for prolonged periods of time. In addition, cell tracking for these prolonged periods can be challenging due to the small size of prokaryotes in general. Future research, therefore, requires the development of tools specifically for anaerobic high-throughput cultivation and imaging purposes. These could include, for example, the development of custom-made anoxic incubators that could be mounted on high-throughput microscopes, together with pipetting robots operating in an oxygen-free atmosphere, the development of anaerobic gloveboxes with greater flexibility for embedding large equipment, and further research on oxygen-independent fluorescent labels. An additional challenge is to further develop culture media for imaging purposes which does not interfere with the visualization due to cloudiness and precipitation of substrates. We have summarized the challenges in anoxic live-imaging and offered potential solutions in Fig. 8.

## CONCLUSIONS

Overcoming the technical and methodological challenges described above would open up new horizons and advance our understanding of anaerobic microorganisms and microbial functions in anoxic environments (Fig. 7). For example, with better anoxic imaging and microfluidic experimentation, we could unravel phenotypic heterogeneity (i.e., differences in phenotype within a clonal population), phenotypic plasticity (i.e., differences in phenotype in response to variation in micro-climate), mutation rates, cellular function, and fitness rates of anaerobic populations which, at this point, are largely unknown. Moreover, these techniques could be integrated in further downflow processes (which may or may not require anoxic cultivation; Figure 6). For example, single-cell RNA sequencing and single-cell genomics or strain-resolved genomics, while still technically challenging, can already be applied to some microbes (134). This gives insights into the transcripts, function, and taxonomy of individual cells. Overall, we hope that this mini-review serves as inspiration for the use of single-cell applications under anoxic conditions. As a community of microbiologists, we need significant investment in anoxic methodological advances. Anaerobic microbiology can be an elitist field as considerable investment is needed to accommodate expensive and bulky equipment. Moreover, experiments are often slower and the reward slower in the current highly competitive academic landscape. This challenge is potentially the reason for the slower developments in innovative single-cell methodologies or why innovative methods are not widely or rapidly adopted (as shown in Fig. 2). To overcome this one avenue is for funding agencies to fund more projects focused on method development although this does not overcome the slower development time.

For individual labs and researchers, our general recommendation to implement single-cell application under anoxic conditions is to give priority to ensure anoxic conditions in their set-up (either cell-sorting or visualization purposes). As it is challenging to get access to commercial anaerobic glove bags due to the high prices, an alternative would be to collaborate with industry to make custom-made boxes, and/or microfluidics devices, as well as to rely on collaborations with labs where these methods are well established. In this way, the resources available are more optimized and it promotes further collaborations with other research groups. Other more long-term solutions could include a global centralized state-of-the-art facility, akin to the diamond labs synchrotron facilities (https://www.diamond.ac.uk/Home.html) where researchers could obtain access through competitive proposals and conduct high-throughput multi-disciplinary anoxic experiments. However, the most tangible option is for microbiologists to come together as an organized community of researchers to reduce the research silos from different disciplines and different expertise areas (e.g., between medical and environmental anaerobic microbiologists). We need initiatives to share resources and protocols, more collaboration avenues, and to design potentially cheaper alternatives to anaerobic gloveboxes (such as 3D printed incubators for anoxic visualization). In addition, we need to work with engineers, chemists, industrial partners, and other researchers to build new anoxic experimentation systems, synthesize new dyes and labels, off the shelf microfluidic systems for prokaryotic cells and other tools. We have created an anoxic cultivation special interest group hosted on Discord which welcomes discussions on how to implement methodological advancements for the benefit of the environment and public health (the invite link is https://discord.gg/Gy5egqkAdV).

## SUPPLEMENTAL MATERIAL

Supplemental file 1 aem.01321-24-s0001.docx List of references for Figure 2.

ASM does not own the copyrights to Supplemental Material that may be linked to, or accessed through, an article. The authors have granted ASM a non-exclusive, world-wide license to publish the Supplemental Material files. Please contact the corresponding author directly for reuse.
