# Supplementary material for: Microbial single-cell applications under anoxic conditions
Source: Appl Environ Microbiol. 2024 Sep 30;90(11):e01321-24. doi: 10.1128/aem.01321-24 (PMC11577760; doi:10.1128/aem.01321-24)
Supplement: Supplemental file 1 — List of references for Figure 2. [file aem.01321-24-s0001.docx]

**MICROBIAL SINGLE-CELL APPLICATIONS UNDER ANOXIC CONDITIONS**

Ciara Keating^1*^, Kerstin Fiege^2*^, Martijn Diender^3,4*^, Diana Z. Sousa^3,4^, Laura Villanueva^2,5^

^1^Department of Engineering, Durham University, Durham DH1 3LE, UK

^2^Department of Marine Microbiology and Biogeochemistry, Royal Netherlands Institute for Sea Research (NIOZ), Texel, Den Burg, The Netherlands

^3^Laboratory of Microbiology, Wageningen University & Research, Wageningen, The Netherlands

^4^Centre for Living Technologies, Alliance TU/e, WUR, UU, UMC Utrecht, Utrecht, The Netherlands

^5^Department of Biology, Utrecht University, Utrecht, The Netherlands

*Shared first authorship. All authors contributed equally. The authorship order was decided by alphabetical first names.

**Corresponding authors:** [ciara.keating@durham.ac.uk](mailto:ciara.keating@durham.ac.uk); [kerstin.fiege@nioz.nl](mailto:kerstin.fiege@nioz.nl); [martijn.diender@wur.nl](mailto:martijn.diender@wur.nl)

**Full list of references for timeline figure**

Schouten S. 1911. Pure cultures from a single cell, isolated under the microscope, p 1910–1911. Konigl. Akad. Wetensch. APSS.

Barber MA. 1914. The pipette method in the isolation of single microorganisms and in the inoculation of substances into living cells: with a technique for dissection, staining and other processes carried out under the higher powers of the microscope. Bureau of Science of the Philippines Government.

Chambers R. 1922. New micromanipulator and methods for the isolation of a single bacterium and the manipulation of living cells. J Infect Dis 31:334–343. https://doi.org/10.1093/infdis/31.4.334

Avery RC, Leland SJ. 1927. A simple method for the isolation of pure cultures from single bacterial cells. J Exp Med 45:1003–1007. https:// doi.org/10.1084/jem.45.6.1003

Huber R, Burggraf S, Mayer T, Barns SM, Rossnagel P, Stetter KO. 1995. Isolation of a hyperthermophilic archaeum predicted by in situ RNA analysis. Nature 376:57–58. https://doi.org/10.1038/376057a0

Raman, C.V. and Krishnan, K.S., 1928. LI. A theory of light-scattering in liquids. *The London, Edinburgh, and Dublin Philosophical Magazine and Journal of Science*, *5*(29), pp.498-512.

Comandon J, Fonbrune P. 1938. Oil chamber for study of living microorganisms, tissue culture and micromanipulation. Ann Inst Pasteur 60

Richter KM. 1948. An improved moist chamber slide for use in micromanipulation. Science 108:192. [https://doi.org/10.1126/science. 108.2799.192](https://doi.org/10.1126/science.%20108.2799.192)

Steinhaus B, Garcia ML, Shen AQ, Angenent LT. 2007. A portable anaerobic microbioreactor reveals optimum growth conditions for the methanogen Methanosaeta concilii. Appl Environ Microbiol 73:1653– 1658. <https://doi.org/10.1128/AEM.01827-06>

Porto, S.P.S. and Wood, D.L., 1962. Ruby optical maser as a Raman source. *Applied Optics*, *1*(101), pp.139-141.

Ashkin A. 1978. Trapping of atoms by resonance radiation pressure. Phys Rev Lett 40:729–732. <https://doi.org/10.1103/PhysRevLett.40.729>

Boye, E., Steen, H.B. and Skarstad, K., 1983. Flow cytometry of bacteria: a promising tool in experimental and clinical microbiology. *Microbiology*, *129*(4), pp.973-980.

Dalterio, R.A., Nelson, W.H., Britt, D. and Sperry, J.F., 1987. An ultraviolet (242 nm excitation) resonance Raman study of live bacteria and bacterial components. *Applied spectroscopy*, *41*(3), pp.417-422.

Fievet A, Ducret A, Mignot T, Valette O, Robert L, Pardoux R, Dolla AR, Aubert C. 2015. Single-cell analysis of growth and cell division of the anaerobe Desulfovibrio vulgaris hildenborough. Front Microbiol 6:1378. <https://doi.org/10.3389/fmicb.2015.01378>

Thompson AW, Crow MJ, Wadey B, Arens C, Turkarslan S, Stolyar S, Elliott N, Petersen TW, van den Engh G, Stahl DA, Baliga NS. 2015. A method to analyze, sort, and retain viability of obligate anaerobic microorganisms from complex microbial communities. J Microbiol Methods 117:74–77. <https://doi.org/10.1016/j.mimet.2015.07.009>

Wakamoto Y, Inoue I, Moriguchi H, Yasuda K. 2001. Analysis of singlecell differences by use of an on-chip microculture system and optical trapping. Fresenius J Anal Chem 371:276–281. https://doi.org/10.1007/ s002160100999

Inoue I, Wakamoto Y, Moriguchi H, Okano K, Yasuda K. 2001. On-chip culture system for observation of isolated individual cells. Lab Chip 1:50–55. <https://doi.org/10.1039/b103931h>

Elfwing A, LeMarc Y, Baranyi J, Ballagi A. 2004. Observing growth and division of large numbers of individual bacteria by image analysis. Appl Environ Microbiol 70:675–678. [https://doi.org/10.1128/AEM.70.2.675- 678.2004](https://doi.org/10.1128/AEM.70.2.675-%20678.2004)

Wang P, Robert L, Pelletier J, Dang WL, Taddei F, Wright A, Jun S. 2010. Robust growth of Escherichia coli. Curr Biol 20:1099–1103. https://doi. org/10.1016/j.cub.2010.04.045

Huh D, Matthews BD, Mammoto A, Montoya-Zavala M, Hsin HY, Ingber DE. 2010. Reconstituting organ-level lung functions on a chip. Science 328:1662–1668. <https://doi.org/10.1126/science.1188302>

Afrizal A, Hitch TCA, Viehof A, Treichel N, Riedel T, Abt B, Buhl EM, Kohlheyer D, Overmann J, Clavel T. 2022. Anaerobic single-cell dispensing facilitates the cultivation of human gut bacteria. Environ Microbiol 24:3861–3881. <https://doi.org/10.1111/1462-2920.15935>

Yin J, Chen X, Li X, Kang G, Wang P, Song Y, Ijaz UZ, Yin H, Huang H. 2022. A droplet-based microfluidic approach to isolating functional bacteria from gut microbiota. Front Cell Infect Microbiol 12:920986. <https://doi.org/10.3389/fcimb.2022.920986>

Bellais S, Nehlich M, Ania M, Duquenoy A, Mazier W, van den Engh G, Baijer J, Treichel NS, Clavel T, Belotserkovsky I, Thomas V. 2022. Speciestargeted sorting and cultivation of commensal bacteria from the gut microbiome using flow cytometry under anaerobic conditions. Microbiome 10:24. <https://doi.org/10.1186/s40168-021-01206-7>
